# Retraction: Inhibition of endothelial cell proliferation and tumor angiogenesis by up-regulating *NDRG2* expression in breast cancer cells

**DOI:** 10.1371/journal.pone.0240574

**Published:** 2020-10-08

**Authors:** The PLOS ONE Editors

After this article [1] was published, concerns were raised about results reported in Figs 1, 3 and 5.

Specifically:

- Similarities were noted between the following western blot data reported in [1] and [2]. In each case, the similar results are used to represent different experiments and/or conditions in the two articles.
  ○ Figure 1A *NDRG2* blot in [1], lanes 1–2, appears similar to the Figure 2D VEGF blot in [2], flipped horizontally.
  ○ Figure1A *NDRG2* blot in [1], lanes 3–5, appears similar to the Figure 2E VEGF blot in [2], flipped horizontally.
  ○ Figure 1A β-actin blot in [1], lanes 3–5, appears similar to the Figure 2E β-actin blot in [2], flipped horizontally.
- The β-actin blot in Fig 1B appears similar to the β-actin blot in Fig 4C for the Normoxia condition.
- The upper left area in Fig 3A, Ad-*NDRG2* panel appears similar to the lower right area of the control panel in Figure 7C of [3], for which copyright is held by the American Cancer Society.
- The following pairs of panels in Fig 5B appear to report overlapping data:
  ○ VHL immunolabelling, Control and Ad-LacZ panels
  ○ HIF-1α immunolabelling, Control and Ad-LacZ panels

The corresponding authors requested retraction and commented that in reviewing the lab records they found that there had been errors in data management and figure preparation.

The underlying data to support this article’s results are no longer available.

In light of the unresolved concerns and in line with the corresponding author’s request, the *PLOS ONE* Editors retract this article.

WL, JZ, and LY agreed with retraction and apologized for the issues with the published article. JM, XY, QW, QZ, YX, HR, LW, YC, SL, and LM either could not be reached or did not respond directly.

Fig 3A, Ad-*NDRG2* panel, reports material similar to a panel in Figure 7C of [3], published in 2012 by the American Cancer Society. The material in [3] is not offered under a CC-BY license and therefore Fig 3A, Ad-*NDRG2* panel is excluded from this article’s [1] license. At the time of retraction, the article [1] was republished to note this exclusion in the Fig 3 legend and the article’s copyright statement.
